# Is Religious Coping a Protective Factor, and for Whom? A Moderation and Subgroup Analysis on Loneliness and Suicide Attempts Among LGBTQ+ Adults Raised as Latter‐Day Saints

**DOI:** 10.1111/sltb.70088

**Published:** 2026-03-31

**Authors:** Seungju Kim, G. Tyler Lefevor, Carson K. Miller, Peter J. Jankowski

**Affiliations:** ^1^ Department of Psychology University of Illinois at Urbana‐Champaign Champaign Illinois USA; ^2^ Psychology Department Utah State University Logan Utah USA; ^3^ The Albert & Jessie Danielsen Institute Boston University Boston Massachusetts USA

**Keywords:** LGBTQ+, loneliness, religious coping, suicide

## Abstract

**Introduction:**

LGBTQ+ adults face elevated rates of loneliness and suicide attempts, yet little research has examined whether religious coping—a protective factor for people generally—effectively mitigates these risks for LGBTQ+ adults raised in theologically conservative, cis/heteronormative religious traditions.

**Method:**

This longitudinal study examined whether religious coping moderates the relationship between loneliness and suicide attempts among 369 LGBTQ+ adults who at some point in their lives were part of the Church of Jesus Christ of Latter‐day Saints, a notably cis/heteronormative tradition, across levels of immoral views of same‐sex sexuality and demographic subgroups.

**Results:**

Contrary to expectations, religious coping generally exacerbated rather than buffered the loneliness‐suicide relationship, with greater religious coping strengthening the loneliness‐suicide relationship among White and transgender/gender‐diverse adults. Religious coping buffered the impact of loneliness on suicide attempts only for adults of Color and non‐religious adults.

**Conclusion:**

These findings challenge assumptions about the universal benefits of religious coping for LGBTQ+ adults raised LDS, suggesting that suicide prevention research should assess individuals' views of the morality of same‐sex sexuality rather than promoting religious coping universally, as this may inadvertently increase risk for certain LGBTQ+ adults in similarly theologically conservative religious contexts.

## 1 Introduction

Despite increased societal acceptance of lesbian, gay, bisexual, transgender, and queer (LGBTQ+) adults, cross‐sectional and longitudinal findings consistently demonstrate broad health disparities across internalized and externalized psychopathological outcomes (Liu et al. 2024; Valen et al. 2025; Wittgens et al. 2022), including acute health outcomes like suicide attempts. Indeed, recent meta‐analytic findings suggest that, compared to cisgender/heterosexual individuals, LGBTQ+ individuals are more likely to experience loneliness (Gorczynski and Fasoli 2022) and almost three to five times more likely to consider or attempt suicide (Hottes et al. 2016; Salway et al. 2019). These disparities can be explained in part by the increased *minority*‐specific stressors experienced by LGBTQ+ individuals (e.g., LGBTQ+ discrimination, internalized LGBTQ+ stigma) (Meyer 2003). Among the sources of minority stress experienced by LGBTQ+ individuals, religion represents a particularly salient yet understudied context. LGBTQ+ adults raised in theologically conservative, cis/heteronormative religious traditions may face unique stressors, including doctrinal condemnation of same‐sex sexuality and gender nonconformity, that compound existing minority stress processes (Lefevor et al. 2023). The present study focuses specifically on LGBTQ+ adults raised in the Church of Jesus Christ of Latter‐day Saints (Latter‐day Saint; LDS), a tradition whose clearly codified cis/heteronormative doctrine makes it an especially informative context for examining religiousness as a protective factor.

Loneliness, defined as the subjective distressing experience that occurs when one's social needs are unmet, regardless of the quantity of social relationships (Peplau and Perlman 1982), serves as a potent risk factor for suicidal behaviors through several well‐established mechanisms. According to Joiner's (2005) interpersonal theory of suicide, suicide attempts occur when three conditions are met: thwarted belongingness (i.e., unmet need for social connection), perceived burdensomeness (i.e., belief that one's death would be worth more than one's life), and acquired capability for suicide (i.e., reduced fear of death and increased pain tolerance). Loneliness directly contributes to thwarted belongingness by creating persistent feelings of social disconnection and isolation, even when surrounded by others (Van Orden et al. 2010).

Indeed, new longitudinal meta‐analytic evidence suggests a robust predictive relationship between loneliness and suicidality (McClelland et al. 2020).

Given the established risks associated with loneliness and its heightened prevalence among LGBTQ+ adults, there is a critical need to identify ways that LGBTQ+ individuals may effectively cope with loneliness to reduce its detrimental effects. While considerable research has focused on risk factors for suicide among LGBTQ+ populations, substantially less attention has been devoted to understanding resilience and coping practices that may mitigate suicide risk in the face of loneliness and other stressors.

For people generally, religiousness (i.e., the search for the divine or sacred in the context of culturally sanctioned rituals or institutions [Harris et al. 2018]), often provides a sense of social connectedness and belonging that may mitigate the negative impacts of loneliness (Gemar 2024). Indeed, in both cross‐sectional and longitudinal studies, religiousness is associated with lower suicide ideation and attempts (Rasic et al. 2011; VanderWeele et al. 2016; Wu et al. 2015). LGBTQ+ adults—particularly the 48% who currently identify as religious in the United States (Alper and Kallo 2025)—may also benefit from religiousness in the face of stressors, with emerging evidence suggesting a slightly positive and largely heterogeneous relationship between religiousness and health among LGBTQ+ people (Lefevor et al. 2021). At the same time, religiousness may also be a source of stigma and oppression for LGBTQ+ people, making it less clear whether leaning on religiousness to combat loneliness will be helpful or harmful for LGBTQ+ people (Lefevor et al. 2023).

LGBTQ+ adults raised LDS in particular face unique barriers due to clearly defined doctrine and practices that discourage gender nonconformity and same‐sex sexual behaviors (Church of Jesus Christ of Latter‐Day Saints 2017). This practice is tied to the belief that sexual orientation and gender identity are linked to sex assigned at birth and appointed by God (Church of Jesus Christ of Latter‐Day Saints 2021). As such, religiousness for currently or formerly LDS adults may serve as an adverse contributor to health through internalized fear of divine punishment or family rejection (Kim et al. 2025). In the present study, we examine the role of religiousness as either a risk or protective factor within LGBTQ+ adults raised LDS, who may represent other LGBTQ+ adults raised in conservative religious traditions.

### 1.1 Religiousness as a Protective Factor Against Suicide

Among cisgender/heterosexual people, religiousness has generally been associated with lower rates of distress, substance misuse, and suicide attempts (Smith et al. 2003). These protective benefits can be attributed through behavioral, social, and psychological pathways according to Koenig (2012). For example, religious people may be at lower risk of experiencing depression (a common predictor of suicide attempts) when they regularly participate in their religious community, which may also provide informational, instrumental, or emotional support. Religious people are also less likely to use or misuse substances in part due to religious and moral beliefs about substances, with moral beliefs being another protective factor of suicide attempts (Jongkind et al. 2019). Finally, religious people may be less likely to experience internalized distress by engaging in unique meaning‐making strategies (i.e., seeking to make meaning in adverse situations [Park 2010]) by seeking the divine or sacred (Park 2007).

Although LGBTQ+ adults are more likely to disaffiliate from religion (Woodell and Schwadel 2020), whereby religious behaviors or social connections are foregone, they may still benefit from leaning on religiousness to cope with life stressors. In his original conceptualization of religious coping, Pargament (1997) describes the practice as an effort to understand or respond to life stressors by relating to the sacred (e.g., God, higher power, enlightenment). When individuals engage in religious coping, they are less likely to report depressive symptoms (Thomas and Barbato 2020) or suicide ideation (Molock et al. 2006). For the present study, religious coping is measured and conceptualized as a “sense of connectedness with a transcendent force, a secure relationship with a caring God, and a belief that life has a greater benevolent meaning” (Pargament et al. 2011, 58). In this way, religious coping may act as a unique buffer against loneliness by providing a sense of connection to the divine in a context where LGBTQ+ adults may feel alone. Moreover, a recent meta‐analysis of sexually diverse individuals suggests that compared to other forms of religiousness, psychological forms (e.g., religious coping) demonstrate one of the strongest positive associations with health (Lefevor et al. 2021). Thus, much like cisgender and heterosexual adults, LGBTQ+ adults may also benefit from religious coping in the face of loneliness.

### 1.2 Religion as a Risk Factor for Suicide

At the same time, LGBTQ+ adults also navigate a more complicated relationship with religiousness compared to cisgender and heterosexual adults. Although more religious traditions and people are increasingly open and affirming toward LGBTQ+ individuals, most religious traditions continue to hold stigmatizing attitudes toward sexual and gender diversity. While religiousness, as a broad construct, demonstrates a decent positive association with health in the general population (Smith et al. 2003), its association with health among LGBTQ+ people is lower (Lefevor et al. 2021). The weaker association between religiousness and health among LGBTQ+ people likely reflects the reality that many LGBTQ+ people experience discrimination and rejection from religious people (Kim et al. 2025; Lefevor et al. 2024) and systems (Todd, Nguyễn, et al. 2025). Further, many LGBTQ+ people internalize cis‐ and hetero‐normative religious teachings, which they then experience as internalized stigma (Lefevor et al. 2023).

For LGBTQ+ adults raised LDS struggling with loneliness, it is thus possible that seeking connection with the divine may lead to enhanced feelings of isolation and more suicide attempts. Religious coping can enhance the risk of suicide attempts when psychological engagement with the divine/sacred exists in the context of tension between religious beliefs and diverse sexual/gender expressions. Indeed, multiple studies demonstrate that religiousness can contribute to increased risk of suicide ideation and attempts when LGBTQ+ adults internalize cis‐ and hetero‐normative teachings about the immorality of same‐sex sexuality (Gibbs and Goldbach 2015; Goodman 2024). As such, connection to the divine/sacred likely represents a consistent source of strength and purpose in life for cisgender and heterosexual adults who may not experience this conflict, whereas for LGBTQ+ adults, connection to the divine may enhance internalized stigma about one's sexual or gender identity, further enhancing the likelihood of suicide attempts.

### 1.3 When and for Whom Might Religion be a Protective or Risk Factor?

Because the relationship between religiousness and health is so heterogeneous for LGBTQ+ people (Lefevor et al. 2021), it is important to wonder *when* (i.e., under what conditions) and *for which* LGBTQ+ people religiousness might be an effective way to cope with loneliness. As noted, religiousness may serve as a stressor or resilience factor for LGBTQ+ people, depending on a variety of factors (Lefevor et al. 2023). One clear answer to the “*when”* question appears to be that religiousness fails to promote health when it fosters internalized stigma through teachings about the immorality of same‐sex sexual behavior. For example, if LGBTQ+ adults believe their sexuality is a fundamental moral flaw, their connection to the Divine/sacred may enhance immoral views of their same‐sex sexuality and further exacerbate stressors (Moffitt and Exline 2025).

The answer to the “*for which*” question is somewhat less clear. LGBTQ+ adults of Color may be more likely to experience the benefits of religious coping than White LGBTQ+ adults. LGBTQ+ adults of Color are much more likely to identify and highly value religion than their White counterparts (Conron et al. 2020). This may be explained in part by how religious communities of Color have served as unique sources of support and belonging for historically minoritized communities (e.g., immigrants, racial/ethnic justice), which may facilitate greater advocacy and openness for LGBTQ+ rights and support. Taken together, religious engagement and coping may thus be more effective for LGBTQ+ adults of color than for White LGBTQ+ adults. Conversely, transgender and gender diverse (TGD) and plurisexual (e.g., bisexual, pansexual) individuals may be less likely to benefit from religious coping than cisgender and monosexual individuals, in part because most religious traditions hold stigmatizing attitudes and values toward gender diverse expressions and plurisexual sexuality (Campbell et al. 2019; Whitley Jr. 2009).

### 1.4 Present Study

With LGBTQ+ adults at higher risk of loneliness and suicide attempts, we attempt to examine how well religious coping may mitigate the detrimental effects of loneliness in a longitudinal sample of LGBTQ+ adults raised religious. Moreover, in addition to probing whether religious coping functions as a protective or risk factor, we also examined the moderating role of views of the morality of same‐sex sexuality in a moderated moderation model. We also performed several moderated moderation models involving multigroup analyses to identify for *whom* it might serve as a protective or risk factor. We examine the two research questions:

1. Does religious coping moderate the effect of loneliness on suicide attempts?
2. Does this effect differ by views of the morality of same‐sex sexuality, race/ethnicity, religious affiliation, sexual identity, or gender identity?

## 2 Methods

### 2.1 Procedures

This study uses data from the Wave I (2020) and Wave II (2022) of an ongoing longitudinal study that looks at religiousness, gender, and sexuality among LGBTQ+ Latter‐day Saints (Lefevor and Skidmore 2025). The longitudinal study has ongoing approval from Utah State University institutional review board (#11707). Participants were recruited to this study in 2020 and again in the spring/summer of 2022 through comprehensive community sampling, including the use of (a) news media stories and advertisements in Utah‐based newspapers, (b) advertisements at therapeutic organizations that serve LGBTQ+ Latter‐day Saints, (c) social media posts in LGBTQ+ Latter‐day Saint centered groups, (d) social media posts from prominent LGBTQ+ Latter‐day Saints, (e) announcements on podcasts that center LGBTQ+ Latter‐day Saints, and (f) snowball sampling. Participants who were part of Wave I were sent three emails inviting them to be part of the second wave. Each participant was compensated $10 at both Wave I and Wave II.

### 2.2 Participants

For participants to be eligible to participate in the overarching longitudinal study, they were required to (a) be at least 18 years old, (b) identify as LGBTQ+, experience some degree of same‐gender sexual attraction, engage in some degree of same‐gender sexual behavior, and/or experience some degree of gender dysphoria, (c) have been baptized a member of the church of Jesus Christ of Latter‐day Saints, (d) live in the United States, and (e) be willing to be contacted by researchers to participate in follow up surveys.

To ensure that participants met the inclusion criteria and to detect fraudulent responses, several checks were included within the survey (e.g., participants answering questions that were identical or near‐identical, responding “somewhat agree” to attention check items). A total of 369 participants completed measures at Wave I. All participants at Wave I were reached out to via email to confirm their identity and willingness to be part of the study (effectively checking for bots). Of those who participated at Wave I, 134 completed measures again at Wave II and were able to be successfully matched to a response from Wave I. The participants were predominantly White, gay, cisgender men who received at least a Bachelor's degree (see Table 1 for more participant demographics).

**TABLE 1:** Participant demographics (*N* = 369).

Characteristic	*N* = 369^a^
Age	
Mean (SD)	31.68 (10.61)
Gender identity	
Cisgender woman	103 (27.91%)
Cisgender man	234 (63.41%)
Transgender woman	7 (1.90%)
Transgender man	5 (1.36%)
Nonbinary	16 (4.34%)
Other	4 (1.08%)
Sexual identity	
Straight no SSA	2 (0.54%)
Straight with SSA	7 (1.90%)
Mostly straight	3 (0.81%)
Bisexual	59 (15.99%)
Mostly gay or lesbian	42 (11.38%)
Gay	140 (37.94%)
Queer	17 (4.61%)
Questioning	5 (1.36%)
Pansexual	53 (14.36%)
Fluid	3 (0.81%)
Asexual	4 (1.08%)
Same‐sex attracted	26 (7.05%)
No label	4 (1.08%)
Other	4 (1.08%)
Racial/ethnic identity	
Asian/Asian American	5 (1.36%)
Black/African American	8 (2.17%)
Latina(o)/Hispanic American	8 (2.17%)
Middle Eastern/Middle Eastern American	4 (1.08%)
Native Hawaiian/Pacific Islander	7 (1.90%)
White/Caucasian/European American	320 (86.72%)
Multiracial/other	17 (4.61%)
Religious identity	
None/unaffiliated	117 (31.71%)
Buddhist	3 (0.81%)
Catholic	17 (4.61%)
Christian—Mainline protestant	25 (6.78%)
Christian—Evangelical or Pentecostal	10 (2.71%)
Jewish	2 (0.54%)
Latter‐day Saint	189 (51.22%)
Other	6 (1.63%)
Education	
High school diploma or GED	23 (6.23%)
Some college	108 (29.27%)
Bachelors	163 (44.17%)
Graduate degree	75 (20.33%)

^a^ *n* (%).

### 2.3 Measures

#### 2.3.1 Covariates

Age and education were treated as continuous, while other demographic variables were collapsed. Gender identities were collapsed into a binary categorical variable: cisgender and trans/gender‐diverse individuals. Sexual identities were collapsed into a binary categorical variable: monosexual (e.g., gay/lesbian, same‐sex attracted) and plurisexual (e.g., bisexual, pansexual). Finally, racial/ethnic identities were collapsed into a binary categorical variable: White and adults of Color (i.e., all other non‐White adults). Religious affiliations were collapsed into a categorical variable: Christians, LDS, None, and Other; however, due to lower cases, the “Other” group was dropped. We categorized Christians separately from Latter‐day Saints because all participants were raised as Latter‐day Saints; thus, identifying as Christian reflects a departure from this natal faith.

#### 2.3.2 Religious Coping

Religious coping was measured using the positive religious coping subscale from the Brief RCOPE, a 14‐item measure (Pargament et al. 2011). Participants were asked to respond to the extent to which they have done each item (e.g., looked for a stronger connection with God; sought God's love and care) within the past month, on a 5‐point Likert scale from *not at all* (1) to *a great deal* (5). Items were averaged, with higher scores indicating higher levels of positive religious coping (α at T1 = 0.96), consistent with prior research demonstrating strong internal reliability (Bourn et al. 2018).

#### 2.3.3 Loneliness

Loneliness was measured using the 6‐item De Jong Gierveld Loneliness Scale (De Jong Gierveld and Van Tilburg 2006). This scale asked participants the extent to which each statement applied to their situation (e.g., there are plenty of people I can rely on when I have problems; there are enough people I feel close to) on a 5‐point Likert scale from *no!* (1) to *yes!* (5). Items were averaged, with higher scores indicating higher levels of loneliness (α at T1 = 0.70), consistent with prior research demonstrating strong internal reliability (Elmer et al. 2022; Hajek et al. 2023).

#### 2.3.4 Suicidality

Past‐year suicidality was measured using the Youth Risk Behavior Survey (Centers for Disease Control and Prevention 2013). Participants responded to the question “During the past 12 months, how many times did you actually attempt suicide?” At Time 1, 9.62% of participants reported at least one suicide attempt, compared to 7.26% at Time 2.

#### 2.3.5 Immoral Views of Same‐Sex Sexuality

Immoral views of same‐sex sexuality were measured using the morality of homosexuality subscale within the Internalized Homonegativity Inventory (Mayfield 2001). This 6‐item subscale asked participants to choose a response that best indicated their current experience as a sexual minority (e.g., I believe it is morally wrong for men to be attracted to each other; in my opinion, homosexuality is harmful to the order of society) on a 6‐point Likert scale from *strongly disagree* (1) to *strongly agree* (6). Items were averaged, with higher scores indicating higher immoral views of same‐sex sexuality (α at T1 = 0.72), consistent with prior research demonstrating strong internal reliability (Sowe et al. 2014).

### 2.4 Analysis Plan

All analyses were performed in *R* (R Core Team 2024) using the *modsem* package (Slupphaug et al. 2024). Regression models were estimated using a maximum likelihood estimator with robust standard errors and a full information maximum likelihood (FIML) estimator to handle missing data. Continuous demographic variables (i.e., age and education) were included as covariates in regression models, while categorical variables (i.e., race/ethnicity, sexual minority identity, and gender identity) were analyzed separately in multigroup models. We employed a hierarchical modeling approach, first estimating a baseline model containing only main effects, followed by a second model that added religious coping, a third model with the 2‐way interaction term (positive religious coping X loneliness), and a final model with the 3‐way interaction term (immoral views of same‐sex sexuality X positive religious coping X loneliness). When the interaction effect was significant, we probed the interaction using the Johnson‐Neyman procedure.

For each subgroup analysis, we estimated both a freely estimated model and a constrained model, in which the interaction effect was constrained to be equal. Differences within subgroups were determined when a chi‐square test suggested the constrained model performed worse (*p* < 0.05) than the freely estimated model (Satorra and Bentler 2001). When the interaction effect within a subgroup was significant, we probed the interaction using the Johnson‐Neyman procedure.

## 3 Results

### 3.1 Preliminary Results

Prior to running the main analyses, we calculated descriptive statistics of focal continuous variables (see Table 2). We also performed a missing data analysis with Little's test (Li 2013) and a visual probe using the *naniar* package (Tierney et al. 2017). Although Little's test suggested data were not missing completely at random (*χ* ^2^ = 74.50, *p* < 0.001), the normed chi‐square was < 3 (*χ* ^2^/df = 2.87; Bollen 1989), and a visual probe using an UpSet plot suggested that the data could be assumed to be missing at random. We also performed an ANOVA to identify if levels of religious coping differed by religious affiliation (see Table S1 and Figure S1).

**TABLE 2:** Descriptive statistics and correlations.

Variable	M	SD	1	2	3	4	5	6
1. T1 Age	31.68	10.61						
2. T1 Education	3.79	0.84	0.47***					
3. T1 Loneliness	2.70	0.73	−0.13*	−0.10				
4. T1 Suicide attempts	0.13	0.47	−0.03	0.06	0.11*			
5. T2 Suicide attempts	0.07	0.25	−0.13	−0.10	0.37***	0.05		
6. T1 Religious coping	2.63	1.28	−0.10*	−0.09	0.03	0.22***	0.29***	
7. T1 Immorality of same‐sex sexuality	2.13	1.29	−0.14**	−0.22***	0.12*	0.31***	0.27**	0.54***

*Note:* *p < 0.05, **p < 0.01, and ***p < 0.001.

### 3.2 Main Analyses

**FIGURE 1:**
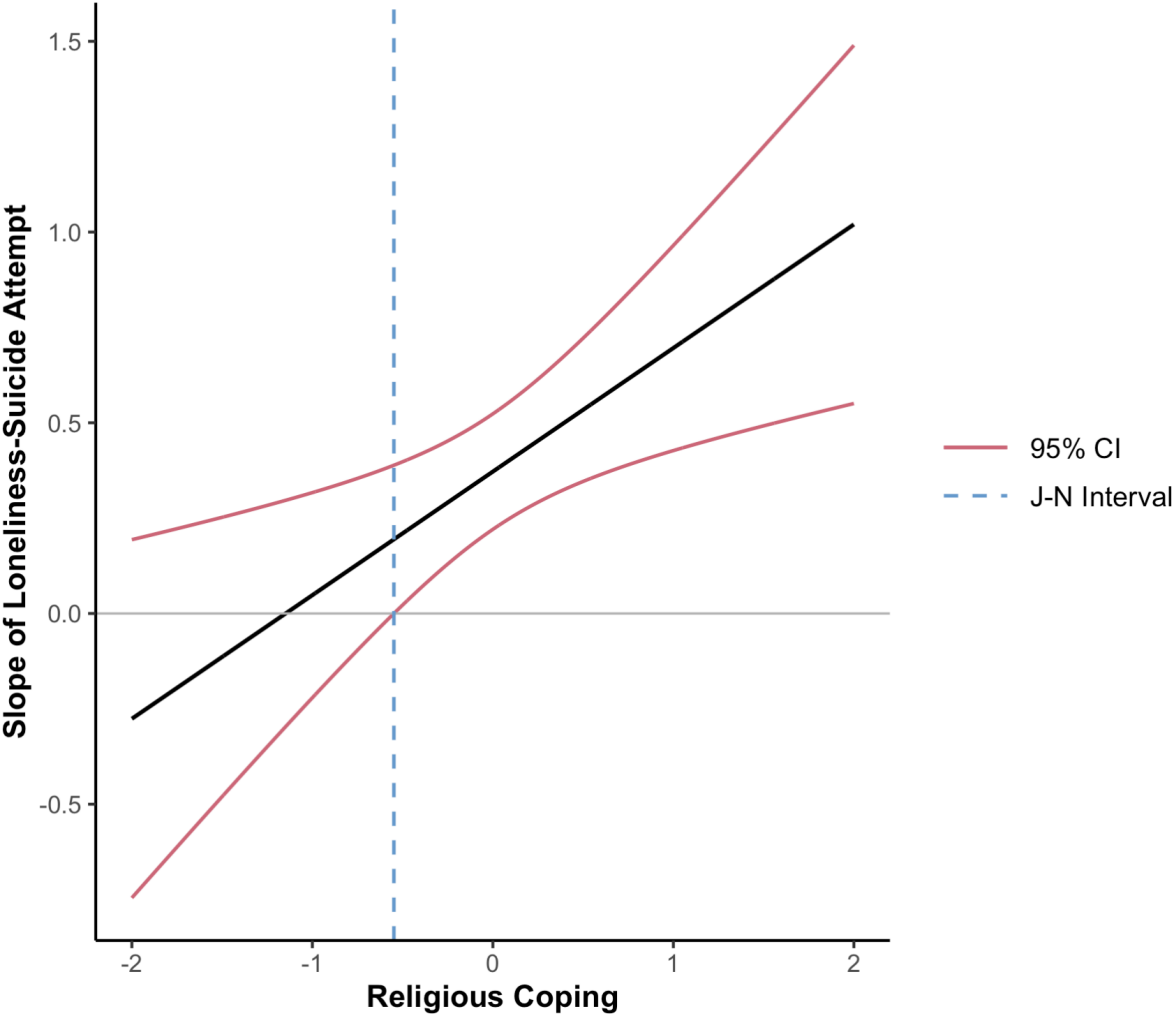
Johnson‐Neyman plot of religious coping.

In the first regression model, we included loneliness, age, education, and suicide attempts at T1 as predictors, with suicide attempts at T2 as the outcome. As expected, results suggest loneliness predicted more suicide attempts (β = 0.32, SE = 0.08, *p* < 0.001). Contrary to expectations, we found that when we added religious coping to the model, both loneliness (β = 0.28, SE = 0.07, *p* < 0.001) and religious coping predicted more suicide attempts (β = 0.24, SE = 0.10, *p* = 0.012). In our final model, we included an interaction term between loneliness and religious coping, which suggested religious coping moderated the effect of loneliness on suicide attempts (β = 0.32, SE = 0.11, *p* = 0.004; see Table 3). Probing the interaction using the Johnson‐Neyman procedure revealed that the effect of loneliness on suicide attempts was significant only at high levels of standardized religious coping (see Figure 1). Specifically, religious coping moderated the slope of loneliness–suicide attempt association when standardized religious coping scores were outside the intervals of −3.79 and −0.55 (*p* < 0.05). However, given the statistical unlikelihood of scores below −3.79 SD, this result meaningfully suggests that religious coping only exacerbated the effect of loneliness on suicide attempts when standardized religious coping scores are greater than (to the right of) –0.55, as depicted on the graph.

**TABLE 3:** Regression models predicting Time 2 suicide attempts.

	First model			Second model			Third model		
	β	SE	*p*	β	SE	*p*	β	SE	*p*
T1 Age	−0.04	0.04	0.246	−0.04	0.04	0.308	−0.03	0.03	0.407
T1 Education	< 0.01	0.09	0.993	< 0.01	0.09	0.961	−0.05	0.1	0.622
T1 Suicide attempt	0.02	0.14	0.898	−0.01	0.15	0.952	< 0.01	0.15	0.999
T1 Loneliness	0.32	0.08	< 0.001	0.28	0.07	< 0.001	0.37	0.08	< 0.001
T1 Religious coping				0.24	0.10	0.012	0.16	0.07	0.030
Loneliness × religious coping							0.32	0.11	0.004
*R* ^2^	0.11			0.15			0.26		

### 3.3 Moderated Moderation Model

#### 3.3.1 Three‐Way Interaction Model

**FIGURE 2:**
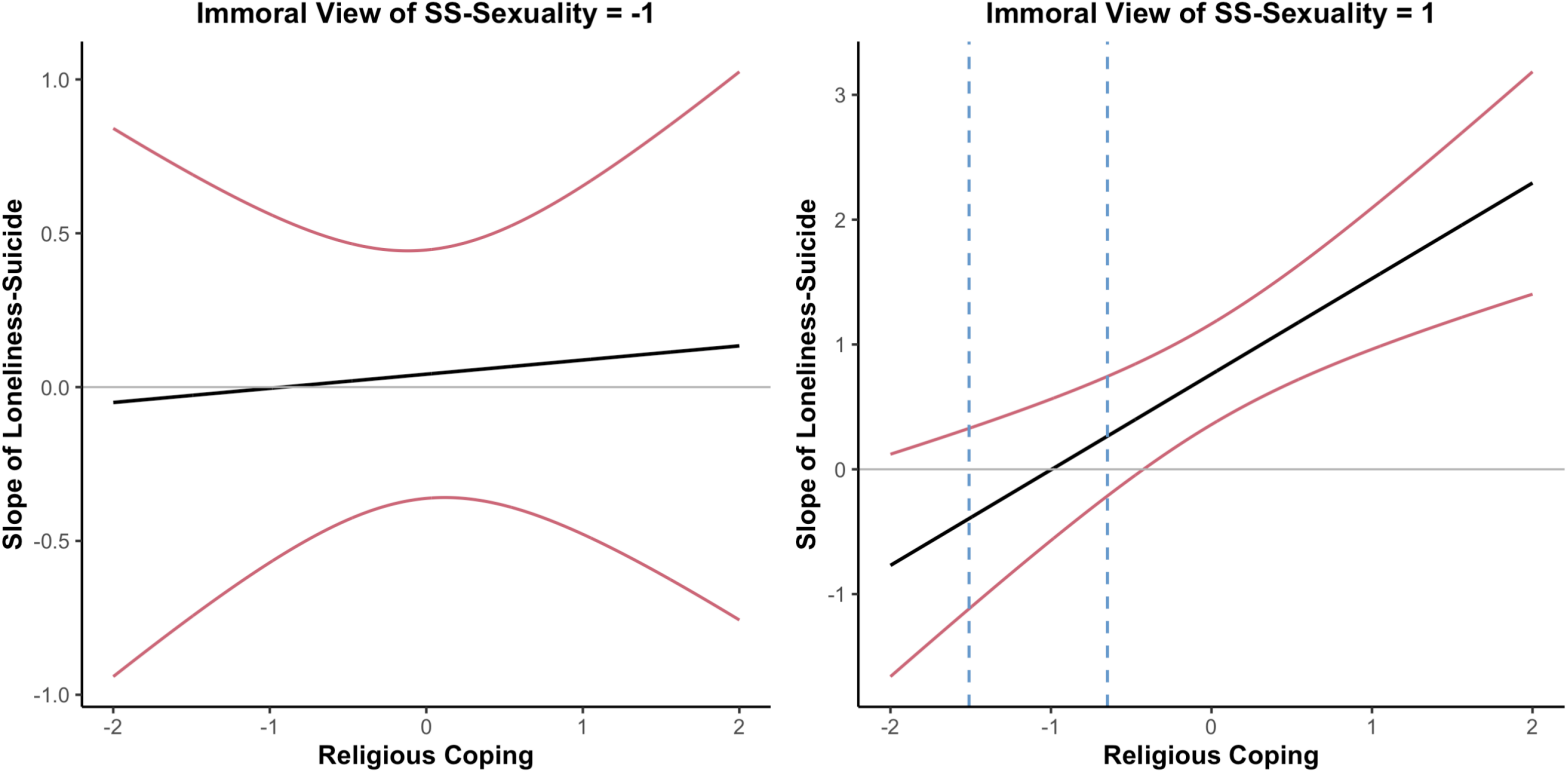
Johnson‐Neyman plot of immoral views of same‐sex sexuality moderated moderation model.

We performed a three‐way interaction between religious coping and loneliness with the morality of same‐sex sexuality. Regression results suggest the morality of same‐sex sexuality moderated the interaction between loneliness and religious coping (β = 0.36, SE = 0.16, *p* = 0.026; see Table 4). Probing the interaction using the Johnson‐Neyman procedure revealed that the effect of immoral views of same‐sex sexuality on the interaction between loneliness and religious coping was significant only at certain levels of immoral views of same‐sex sexuality (see Figure 2). Specifically, at high levels of standardized immoral views of same‐sex sexuality (+1 SD), religious coping moderated the slope of the loneliness‐religious coping interaction when standardized religious coping scores were outside the intervals of −1.51 and −0.65 (*p* < 0.05). In other words, at high levels of immoral views of same‐sex sexuality, religious coping exacerbated the effect of loneliness on suicide attempts at average or higher levels of religious coping (above −0.65 SD), while beyond the lower boundary (−1.51 SD), the effect was negative. However, the lower values represent a small percentage of the sample that could suggest no meaningful buffering effect.

**TABLE 4:** Moderated moderation model predicting Time 2 suicide attempt.

	β	SE	*p*
T1 Age	0.01	0.14	0.920
T1 Education	−0.02	0.03	0.458
T1 Suicide attempt	−0.01	0.09	0.907
T1 Loneliness	0.14	0.07	0.038
T1 Religious coping	0.07	0.09	0.423
T1 Immoral views of same‐sex sexuality	0.17	0.15	0.263
Loneliness × religious coping	0.40	0.12	< 0.001
Loneliness × religious coping × immoral views of same‐sex sexuality	0.36	0.16	0.026
*R* ^2^	0.41		

However, at low levels of standardized immoral views of same‐sex sexuality (−1 SD), religious coping did not moderate the slope of loneliness‐religious coping interaction at any level. In other words, at low levels of immoral views of same‐sex sexuality, religious coping had no meaningful buffering or exacerbating effect.

#### 3.3.2 Subgroup Analyses

**FIGURE 3:**
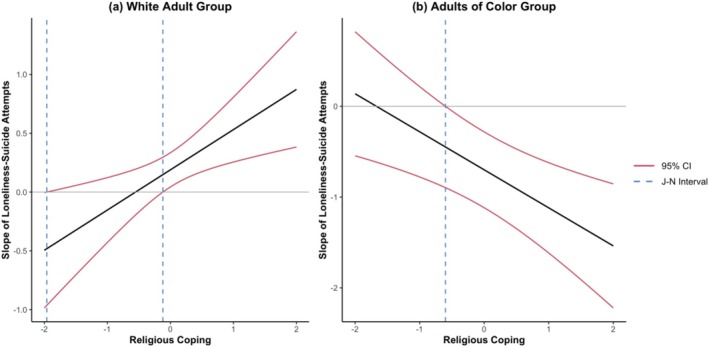
Johnson‐Neyman plot of adults by race/ethnicity.

We performed four multigroup analyses for race/ethnicity, sexual identity, gender identity, and religious affiliation. A chi‐square test of differences suggested the 2‐way interaction effect involving religious coping X loneliness differed between White adults and adults of Color (Δ*χ* ^2^[1] = 8.13, *p* = 0.004). Regression results suggested that religious coping significantly moderated the association between loneliness and suicide attempts for both White adults (β = 0.34, SE = 0.12, *p* = 0.004) and adults of Color (β = −0.42, SE = 0.13, *p* = 0.002). Using the Johnson‐Neyman procedure to probe these interactions revealed distinct patterns by race/ethnicity (see Figure 3). Similar to the overall sample, religious coping moderated the slope of loneliness–suicide attempt association when standardized religious coping scores were outside the intervals of −1.96 and −0.12 (*p* < 0.05) for White adults (see Figure 3a). In other words, religious coping exacerbated the effect of loneliness on suicide attempts at average or higher levels of religious coping (above −0.12 SD), while the lower boundary (−1.96 SD) represents a small percentage of the sample that could suggest no meaningful buffering effect. In contrast, religious coping moderated the slope of loneliness–suicide attempt association when standardized religious coping scores were above (to the right of) −0.60 (*p* < 0.05) for adults of Color (see Figure 3b). In other words, religious coping buffered the effect of loneliness on suicide attempts at higher levels of religious coping (above −0.60 SD).

Next, a chi‐square test of differences suggested the interaction effect did not differ between monosexual and plurisexual adults (Δ*χ* ^2^[1] = 0.36, *p* = 0.549).

**FIGURE 4:**
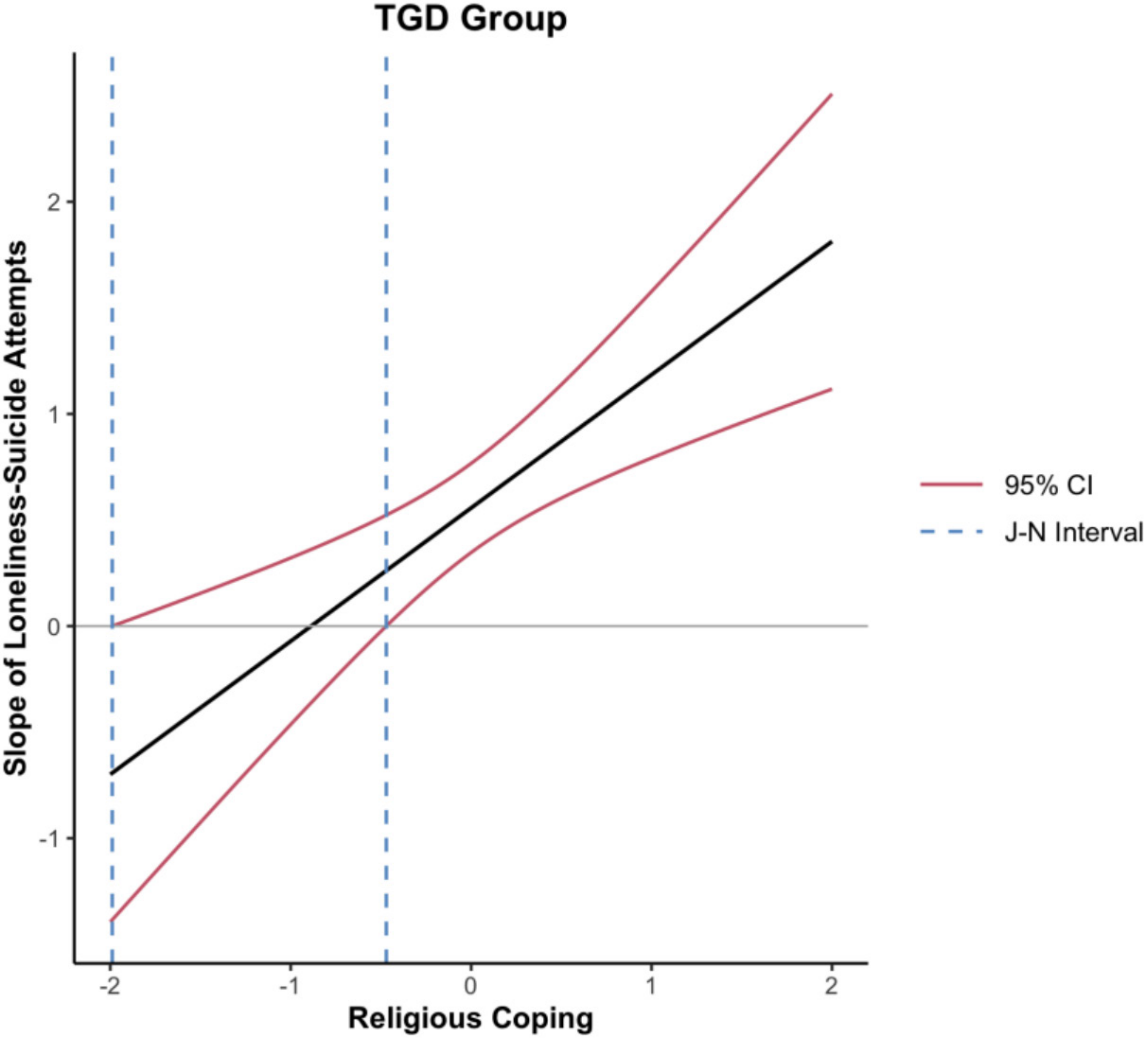
Johnson‐Neyman plot of trans/gender‐diverse adults.

A chi‐square test of differences suggested the interaction effect differed between cisgender and TGD adults (Δ*χ* ^2^[1] = 8.47, *p* = 0.004). Regression results suggested that religious coping significantly moderated the association between loneliness and suicide attempts for TGD adults (β = 0.69, SE = 0.21, *p* = 0.001), but not for cisgender adults (β = −0.09, SE = 0.13, *p* = 0.496). Probing the interaction using the Johnson‐Neyman procedure revealed that for TGD individuals (see Figure 4), religious coping moderated the slope of loneliness–suicide attempt association when standardized religious coping scores were outside the intervals of −1.99 and −0.47 (*p* < 0.05). In other words, religious coping exacerbated the effect of loneliness on suicide attempts at average or higher levels of religious coping (above −0.47 SD), while the lower boundary (−1.99 SD) represents a small percentage of the sample that could suggest no meaningful buffering effect.

**FIGURE 5:**
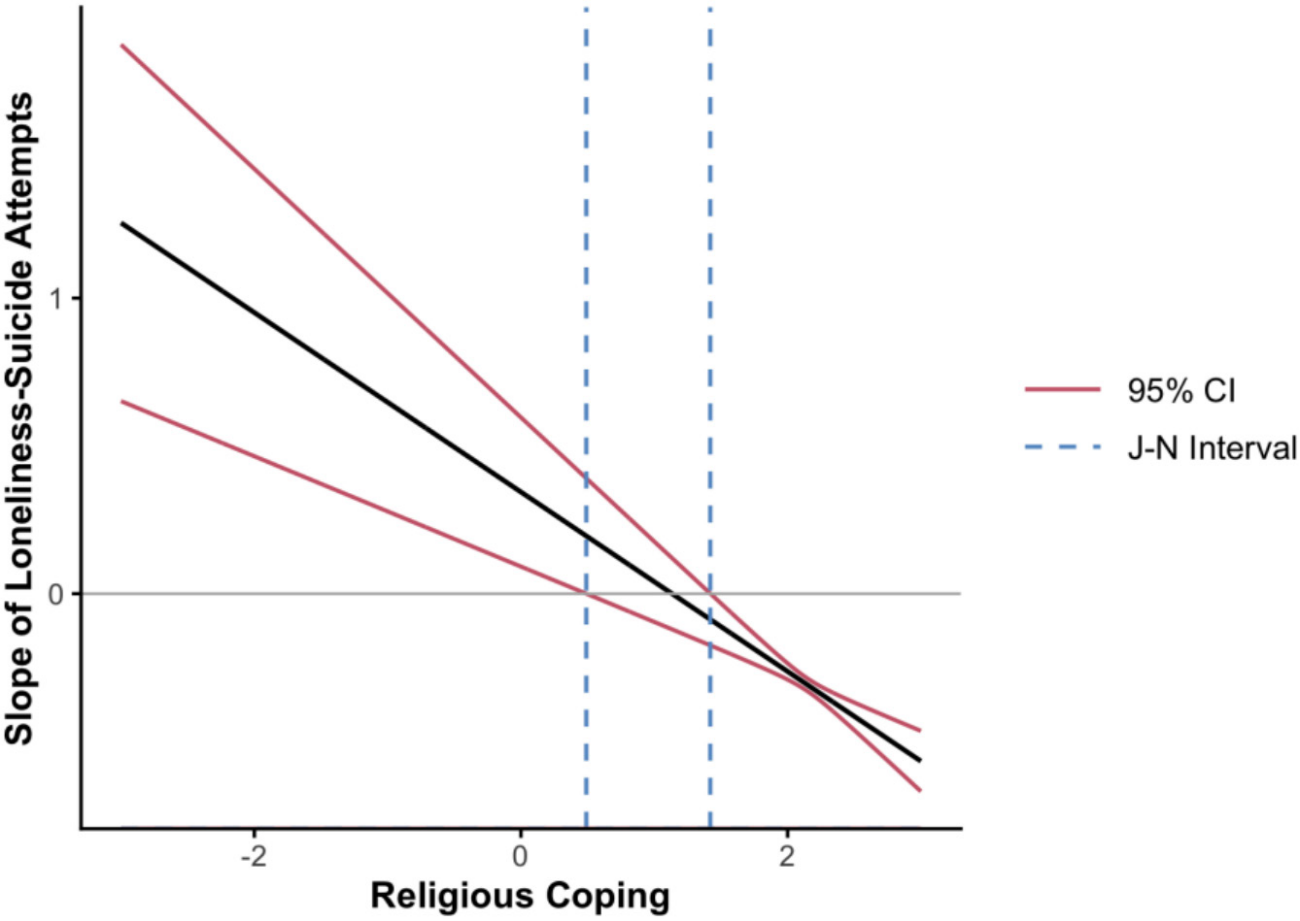
Johnson‐Neyman plot of non‐religious adults.

Finally, a chi‐square test of differences suggested the interaction effect differed by religious affiliation (Δ*χ* ^2^[2] = 24.62, *p* < 0.001). Regression results suggested that religious coping significantly moderated the association between loneliness and suicide attempts for religious Nones (β = −0.30, SE = 0.12, *p* = 0.013), but not for LDS adults (β < 0.01, SE = 0.04, *p* = 1.000) nor Christian adults (β = 0.36, SE = 0.36, *p* = 0.319). Probing the interaction using the Johnson‐Neyman procedure revealed that for religious Nones, religious coping moderated the slope of loneliness–suicide attempt association when standardized religious coping scores were outside the intervals of 0.49 and 1.42 (*p* < 0.05) (see Figure 5). In other words, religious coping buffered the effect of loneliness on suicide attempts at low levels (below 0.49 SD) and high levels (above 1.42 SD) of religious coping. To contextualize why non‐religious individuals might benefit from religious coping, we performed an exploratory bivariate ANOVA between religious affiliation and immoral views of same‐sex sexuality. An omnibus ANOVA reveals a significant association (*F*[2, 355] = 168.21, *p* < 0.001); post hoc Tukey's pairwise tests reveal that while Christians and LDS adults did not differ (*d* = 0.17, 95% CI [−0.135, 0.481]), Nones reported lower levels of immoral views of same‐sex sexuality than Christian (*d* = 2.20, 95% CI [1.833, 2.564]) and LDS (*d* = 2.03, 95% CI [1.750, 2.301]) adults.

## 4 Discussion

The present study examined whether religious coping moderates the relationship between loneliness and suicide attempts among LGBTQ+ adults raised as LDS, who represent a subpopulation of LGBTQ+ adults raised in a conservatively religious context, and whether this moderation differs across immoral views of same‐sex sexuality and demographic subgroups. Our findings reveal a complex and nuanced picture that challenges assumptions about the protective benefits of religious coping. Contrary to expectations, religious coping generally exacerbated rather than mitigated the relationship between loneliness and suicide attempts among LGBTQ+ adults. However, important variations emerged across levels of immoral views of same‐sex sexuality, racial/ethnic groups, gender identity, and religious affiliation, highlighting the potential heterogeneous nature of religiousness on health among LGBTQ+ people with a conservative religious background.

### 4.1 Religious Coping Exacerbates the Effect of Loneliness on Suicide Attempts

Although we considered religious coping as potentially beneficial for LGBTQ+ adults, our findings suggest an exacerbating effect instead. In fact, not only did religious coping exacerbate the impact of loneliness, but it also predicted higher suicide attempts on its own. As such, the clearest first takeaway of our work is that religious coping may largely not be an effective coping mechanism for LGBTQ+ adults. Prior scholars have postulated how religiousness may act as a stressor or resilience factor under unique conditions (Lefevor et al. 2023, 2024) for LGBTQ+ people, such as when their religion is affirming. It is possible that when LGBTQ+ adults perceive the Divine/sacred to affirm all or key aspects of their personhood, they may be more likely to benefit from religiousness, a reason why LGBTQ+ adults raised in theologically conservative, cis/heteronormative religious traditions may experience religious coping as a stressor.

Likewise, when LGBTQ+ adults hold views that same‐sex sexuality is immoral, they may be less likely to benefit from religiousness, as supported by our findings. These outcomes highlight how specific psychological processes may determine whether connecting with the Divine/sacred proves helpful or harmful for LGBTQ+ adults. Beyond the immoral views of same‐sex sexuality, the degree of identity conflict or integration between religious and sexual/gender identities represents another critical psychological factor that may shape the complex relationship between religiousness and mental health outcomes in this population.

Our subgroup analyses further illuminate how these harmful effects of religious coping may be unevenly distributed across LGBTQ+ people, with certain groups of LGBTQ+ people experiencing enhanced risk. Notably, while White LGBTQ+ adults reported religious coping as an exacerbating effect, LGBTQ+ adults of Color demonstrated a buffering effect. White adults may be less likely to benefit from religious coping, in part due to broader demographic trends toward decreased religious identification and lower religious salience among this population (Conron et al. 2020). When religious coping is employed by individuals with weaker religious foundations or conflicted religious identities, it may lack the protective benefits that would otherwise buffer against psychological distress.

Similarly, we found that TGD adults reported a relatively high exacerbating effect compared to cisgender adults. TGD individuals navigate uniquely challenging pathways when engaging with religiousness, as they continue to face greater societal minoritization compared to cisgender sexual minority individuals who have experienced increased acceptance in recent decades. This heightened marginalization may intensify the psychological conflict inherent in seeking connection with the Divine/sacred. Indeed, Exline and colleagues (2021) found that TGD individuals were more likely to disaffiliate and disengage with religion, which likely contributes to psychological conflict when attempting to connect with or seek comfort from the Divine/sacred through religious coping.

### 4.2 Does Anyone Benefit From Religious Coping?

While our overall findings demonstrate that religious coping generally exacerbates the relationship between loneliness and suicide attempts among LGBTQ+ adults, an important exception emerged that reveals *for whom* religious coping may be beneficial: adults of Color and non‐religious individuals. Indeed, adults of Color benefited from religious coping; this pattern may reflect the distinctive role that religiousness plays within many communities of Color, who are more likely to identify as religious (Conron et al. 2020), and where religion often serves as a source of cultural identity, community support, and historical resilience in the face of systemic oppression. For LGBTQ+ individuals of Color, religiousness may offer established frameworks for navigating multiple marginalized identities and accessing community resources that transcend individual spiritual practice.

We also found that when non‐religious individuals (i.e., those who do not affiliate with organized religion) engaged in religious coping, the association between loneliness and suicide became weaker. Although seemingly counterintuitive, we propose two likely reasons why. First, because immoral views of same‐sex sexuality are significantly (i.e., large effect size) lower in non‐religious compared to religious (i.e., LDS and Christians) individuals, non‐religious adults may be more likely to benefit from their search for a “sense of connectedness with a transcendent force, a secure relationship with a caring God, and a belief that life has a greater benevolent meaning” (Pargament et al. 2011, 58). Indeed, as demonstrated by findings in the present study, higher immoral views of same‐sex sexuality exacerbated the negative effect of religious coping on the loneliness–suicide relationship. Having left organized religion and its doctrines, non‐religious individuals may be less likely to hold views that devalue or stigmatize their sexuality, in contrast to religious adults who may continue to affiliate with organized religion and their doctrines, which view same‐sex sexuality as immoral. Second, emerging evidence suggests LGBTQ+ adults who leave religion continue to engage with some aspects of religion or spirituality (Todd, Watanabe, and Blackburn 2025). As such, when LGBTQ+ adults willingly engage with religiousness or spirituality detached from organized religion, they may be more likely to benefit from their own construction of the search for the sacred.

### 4.3 Limitations and Future Directions

Although we used a large longitudinal sample of LGBTQ+ adults raised religious, we acknowledge several limitations of the present study. First, while we identified specific conditions under which religious coping may benefit or harm LGBTQ+ adults, we did not identify the underlying mechanisms that facilitate these outcomes. Understanding these mechanisms is crucial for developing targeted interventions that can either identify the protective aspects of religious coping or provide alternative strategies for those at risk of harm. Second, although we demonstrated moderating effects using longitudinal data, our study utilized only two timepoints, which limits our ability to establish robust causal inferences and examine how these relationships may evolve over time. Multiple timepoints would allow for a better understanding of whether these effects persist, intensify, or diminish as individuals navigate their religious and sexual/gender identities across the lifespan. Third, although significant subgroup differences emerged, future studies should seek to replicate these findings in larger, more racially and ethnically diverse samples to better understand how cultural context shapes the relationship between religious coping, loneliness, and suicide risk among LGBTQ+ adults. Our modestly sized sample of White LGBTQ+ adults raised as LDS may not capture the full gender/sexual/racial diversity among LGBTQ+ adults across the United States (much less other contexts). Our study focused on LGBTQ+ adults raised as LDS to represent other theologically conservative and cis/heteronormative religious contexts. Future research should also use LGBTQ+ adults raised in other religious traditions who may be more affirming.

Fourth, detecting interaction effects requires substantially larger sample sizes than detecting main effects to achieve adequate statistical power (Gelman et al. 2021). As such, the two‐ and three‐way interactions reported in the present study may be noisier and less reliable than the main effects and should be interpreted with appropriate caution. Fifth, future research should examine whether these patterns replicate when using suicidal ideation rather than suicide attempts as the outcome. Suicidal ideation is more prevalent and represents an earlier point in the suicidality continuum, meaning religious coping may operate differently at this stage. Understanding whether religious coping buffers or exacerbates the loneliness‐ideation relationship could inform earlier intervention efforts. Last, our results may have been affected by missing data, particularly in the context of unequal subgroup sizes for the multigroup analyses. We did use FIML estimation, an advanced missing data technique appropriate to the MAR mechanism for missingness. Nevertheless, future research might benefit from greater participation across waves of data.

## 5 Conclusion

Together, the findings of our study suggest that religious coping may overall exacerbate the effects of loneliness on LGBTQ+ adults raised as LDS or among LGBTQ+ individuals raised in similar theologically conservative, cis/heteronormative religious contexts, with some exceptions for adults of Color and those with low immoral views of same‐sex sexuality. These results challenge prevailing assumptions about the universal benefits of religious coping and underscore the critical importance of psychological processes in determining when religiousness serves as a source of strength versus distress for LGBTQ+ individuals. These findings have significant implications for suicide prevention efforts, suggesting that traditional interventions encouraging religious coping as a universal protective factor may inadvertently increase risk for certain subgroups, while tailored approaches that assess individual levels of religious‐sexual identity conflict may prove more effective. Future research should continue exploring the mechanisms underlying these differential effects to develop targeted interventions that acknowledge the complex, heterogeneous relationship between religiousness and mental health among LGBTQ+ adults.

## Author Contributions

**Seungju Kim:** conceptualization, formal analysis, investigation, methodology, writing – original draft, writing – review and editing. **G. Tyler Lefevor:** data curation, funding acquisition, supervision, writing – original draft, writing – review and editing. **Carson K. Miller:** writing – original draft, writing – review and editing. **Peter J. Jankowski:** methodology, supervision, writing – review and editing.

## Funding

Data collection was supported by startup funding from Rhodes College and Utah State University. This material is based upon work supported by the National Science Foundation Graduate Research Fellowship Program under Grant No. DGE 21‐46756 to SK. Any opinions, findings, and conclusions or recommendations expressed in this material are those of the author(s) and do not necessarily reflect the views of the National Science Foundation.

## Ethics Statement

Informed consent was obtained from all participants included in the study. The longitudinal study was conducted in accordance with study protocols approved by the Institutional Review Board at Utah State University (#11707).

## Conflicts of Interest

The authors declare no conflicts of interest.

## Supporting information


**Appendix S1:** sltb70088‐sup‐0001‐AppendixS1.docx.

## Data Availability

Because participants did not consent to the release of their data, it will not be made publicly available. It may be available upon reasonable request after signing a written agreement by contacting the second author.

## References

[sltb70088-bib-0001] Alper, B. A. , and A. Kallo . 2025. Religion and Spirituality Among LGBT Americans. Pew Research Center. https://www.pewresearch.org/short‐reads/2025/08/22/religion‐and‐spirituality‐among‐lgbt‐americans/.

[sltb70088-bib-0053] Bollen, K. A. 1989. Structural Equations with Latent Variables. 1st ed. Wiley. 10.1002/9781118619179.

[sltb70088-bib-0002] Bourn, J. R. , K. A. Frantell , and J. R. Miles . 2018. “Internalized Heterosexism, Religious Coping, and Psychache in LGB Young Adults Who Identify as Religious.” Psychology of Sexual Orientation and Gender Diversity 5, no. 3: 303–312. 10.1037/sgd0000274.

[sltb70088-bib-0003] Campbell, M. , J. D. X. Hinton , and J. R. Anderson . 2019. “A Systematic Review of the Relationship Between Religion and Attitudes Toward Transgender and Gender‐Variant People.” International Journal of Transgenderism 20, no. 1: 21–38. 10.1080/15532739.2018.1545149. 32999592 PMC6830999

[sltb70088-bib-0055] Centers for Disease Control and Prevention (CDC) . 2013. “Youth risk behavior survey (YRBS).”

[sltb70088-bib-0004] Church of Jesus Christ of Latter‐Day Saints . 2017. “Young Single Adult Face to Face With Elder Oaks and Elder Ballard: What Can Homosexuals Do to Stay Firm in the Gospel?” https://www.lds.org/broadcasts/face‐to‐face/oaks‐ballard?lang=eng.

[sltb70088-bib-0005] Church of Jesus Christ of Latter‐Day Saints . 2021. “What Does Transgender Mean?” https://churchofjesuschrist.org/topics/transgender/understanding‐yourself.

[sltb70088-bib-0006] Conron, K. J. , S. K. Goldberg , and K. O'Neill . 2020. Religiosity Among LGBT Adults in the US, 27. Williams Institute. https://williamsinstitute.law.ucla.edu/publications/lgbt‐religiosity‐us/.

[sltb70088-bib-0007] Elmer, E. M. , T. van Tilburg , and T. Fokkema . 2022. “Minority Stress and Loneliness in a Global Sample of Sexual Minority Adults: The Roles of Social Anxiety, Social Inhibition, and Community Involvement.” Archives of Sexual Behavior 51, no. 4: 2269–2298. 10.1007/s10508-021-02132-3. 35084615 PMC9192366

[sltb70088-bib-0054] Exline, J. J. , A. Przeworski , E. K. Peterson , M. R. Turnamian , N. Stauner , and A. Uzdavines . 2021. “Religious and Spiritual Struggles among Transgender and Gender‐Nonconforming Adults.” Psychology of Religion and Spirituality 13, no. 3: 276–286. 10.1037/rel0000404.

[sltb70088-bib-0008] Gelman, A. , J. Hill , and A. Vehtari . 2021. Regression and Other Stories. Cambridge University Press.

[sltb70088-bib-0049] Gemar, A. 2024. “Religion and Loneliness: Investigating Different Aspects of Religion and Dimensions of Loneliness.” Religion 15, no. 4: 488. 10.3390/rel15040488.

[sltb70088-bib-0009] Gibbs, J. J. , and J. Goldbach . 2015. “Religious Conflict, Sexual Identity, and Suicidal Behaviors Among LGBT Young Adults.” Archives of Suicide Research 19, no. 4: 472–488. 10.1080/13811118.2015.1004476. 25763926 PMC4706071

[sltb70088-bib-0051] Gierveld, J. D. J. , and T. Van Tilburg . 2006. “A 6‐Item Scale for Overall, Emotional, and Social Loneliness: Confirmatory Tests on Survey Data.” Research on Aging 28, no. 5: 582–598. 10.1177/0164027506289723.

[sltb70088-bib-0010] Goodman, M. A. 2024. “Associations Between Religion and Suicidality for LGBTQ Individuals: A Systematic Review.” Archive for the Psychology of Religion 46, no. 2: 157–179. 10.1177/00846724241235181.

[sltb70088-bib-0011] Gorczynski, P. , and F. Fasoli . 2022. “Loneliness in Sexual Minority and Heterosexual Individuals: A Comparative Meta‐Analysis.” Journal of Gay & Lesbian Mental Health 26, no. 2: 112–129. 10.1080/19359705.2021.1957742.

[sltb70088-bib-0012] Hajek, A. , H.‐H. König , M. Blessmann , and K. Grupp . 2023. “Loneliness and Social Isolation Among Transgender and Gender Diverse People.” Healthcare 11, no. 10: 1517. 10.3390/healthcare11101517. 37239802 PMC10217806

[sltb70088-bib-0013] Harris, K. A. , D. S. Howell , and D. W. Spurgeon . 2018. “Faith Concepts in Psychology: Three 30‐Year Definitional Content Analyses.” Psychology of Religion and Spirituality 10, no. 1: 1–29. 10.1037/rel0000134.

[sltb70088-bib-0014] Hottes, T. S. , L. Bogaert , A. E. Rhodes , D. J. Brennan , and D. Gesink . 2016. “Lifetime Prevalence of Suicide Attempts Among Sexual Minority Adults by Study Sampling Strategies: A Systematic Review and Meta‐Analysis.” American Journal of Public Health 106, no. 5: e1–e12. 10.2105/AJPH.2016.303088. PMC4985071 27049424

[sltb70088-bib-0015] Joiner, T. 2005. Why People Die by Suicide. Harvard University Press. 10.2307/j.ctvjghv2f.

[sltb70088-bib-0050] Jongkind, M. , B. van den Brink , H. Schaap‐Jonker , N. van der Velde , and A. W. Braam . 2019. “Dimensions of Religion Associated with Suicide Attempt and Suicide Ideation in Depressed, Religiously Affiliated Patients.” Suicide and Life‐threatening Behavior 49, no. 2: 505–519. 10.1111/sltb.12456. 29676507

[sltb70088-bib-0016] Kim, S. , G. T. Lefevor , and S. J. Skidmore . 2025. “Mitigating the Impact of Religiously Based Family Expectations on Depression Among Sexual and Gender Minorities: The Role of Authenticity.” Journal of Homosexuality 72, no. 8: 1401–1425. 10.1080/00918369.2024.2378745. 39028857

[sltb70088-bib-0017] Koenig, H. G. 2012. “Religion, Spirituality, and Health: The Research and Clinical Implications.” ISRN Psychiatry 2012: 278730. 10.5402/2012/278730. 23762764 PMC3671693

[sltb70088-bib-0018] Lefevor, G. T. , E. B. Davis , J. Y. Paiz , and A. C. P. Smack . 2021. “The Relationship Between Religiousness and Health Among Sexual Minorities: A Meta‐Analysis.” Psychological Bulletin 147, no. 7: 647–666. 10.1037/bul0000321.supp. 33793286

[sltb70088-bib-0019] Lefevor, G. T. , C. Etengoff , E. B. Davis , et al. 2023. “Religion/Spirituality, Stress, and Resilience Among Sexual and Gender Minorities: The Religious/Spiritual Stress and Resilience Model.” Perspectives on Psychological Science 18, no. 6: 17456916231179137. 10.1177/17456916231179137. 37369080

[sltb70088-bib-0020] Lefevor, G. T. , S. Kim , and A. M. Perez‐Figueroa . 2024. “How Does Religiousness Influence Health Among Sexual and Gender Minorities? Evaluating the Propositions of the Religious/Spiritual Stress and Resilience Model.” Psychology of Sexual Orientation and Gender Diversity. 10.1037/sgd0000749.

[sltb70088-bib-0058] Lefevor, G. T. , and S. J. Skidmore . 2025. “Navigating Faith Transitions: A 4‐Year Longitudinal Examination of Religious Deidentification among LGBTQ+ Latter‐Day Saints.” Journal of Counseling Psychology (Washington, US) 72, no. 1: 15–29. 10.1037/cou0000765. 39388138

[sltb70088-bib-0021] Li, C. 2013. “Little's Test of Missing Completely at Random.” Stata Journal 13, no. 4: 795–809. 10.1177/1536867X1301300407.

[sltb70088-bib-0022] Liu, M. , V. R. Patel , S. L. Reisner , and A. S. Keuroghlian . 2024. “Health Status and Mental Health of Transgender and Gender‐Diverse Adults.” JAMA Internal Medicine 184, no. 8: 984–986. 10.1001/jamainternmed.2024.2544. 38913367 PMC11197009

[sltb70088-bib-0052] Mayfield, W. 2001. “The Development of an Internalized Homonegativity Inventory for Gay Men.” Journal of Homosexuality 41, no. 2: 53–76. 10.1300/J082v41n02_04. 11482428

[sltb70088-bib-0023] McClelland, H. , J. J. Evans , R. Nowland , E. Ferguson , and R. C. O'Connor . 2020. “Loneliness as a Predictor of Suicidal Ideation and Behaviour: A Systematic Review and Meta‐Analysis of Prospective Studies.” Journal of Affective Disorders 274: 880–896. 10.1016/j.jad.2020.05.004. 32664029

[sltb70088-bib-0024] Meyer, I. H. 2003. “Prejudice, Social Stress, and Mental Health in Lesbian, Gay, and Bisexual Populations: Conceptual Issues and Research Evidence.” Psychological Bulletin 129, no. 5: 674–697. 10.1037/0033-2909.129.5.674. 12956539 PMC2072932

[sltb70088-bib-0048] Moffitt, A. C. , and J. J. Exline . 2025. “Torn at the Seams: Moral Struggles Surrounding Same‐Sex Relationships in Christian‐Raised Individuals Reporting Same‐Sex Attraction.” Psychology of Religion and Spirituality, ahead of print, April 28. 10.1037/rel0000564.

[sltb70088-bib-0025] Molock, S. D. , R. Puri , S. Matlin , and C. Barksdale . 2006. “Relationship Between Religious Coping and Suicidal Behaviors Among African American Adolescents.” Journal of Black Psychology 32, no. 3: 366–389. 10.1177/0095798406290466. 17080183 PMC1630686

[sltb70088-bib-0026] Pargament, K. , M. Feuille , and D. Burdzy . 2011. “The Brief RCOPE: Current Psychometric Status of a Short Measure of Religious Coping.” Religion 2, no. 1: 51–76. 10.3390/rel2010051.

[sltb70088-bib-0027] Pargament, K. I. 1997. The Psychology of Religion and Coping: Theory, Research, Practice. Guilford Press.

[sltb70088-bib-0028] Park, C. L. 2007. “Religiousness/Spirituality and Health: A Meaning Systems Perspective.” Journal of Behavioral Medicine 30, no. 4: 319–328. 10.1007/s10865-007-9111-x. 17522971

[sltb70088-bib-0029] Park, C. L. 2010. “Making Sense of the Meaning Literature: An Integrative Review of Meaning Making and Its Effects on Adjustment to Stressful Life Events.” Psychological Bulletin 136, no. 2: 257–301. 10.1037/a0018301. 20192563

[sltb70088-bib-0030] Peplau, L. A. , and D. Perlman . 1982. “Perspectives on Loneliness.” In Loneliness: A Sourcebook of Current Theory, Research and Therapy, 1–18. Wiley.

[sltb70088-bib-0031] R Core Team . 2024. R: A Language and Environment for Statistical Computing. (Version 4.3.3) [R Programming]. R Foundation for Statistical Computing. https://www.R‐project.org/.

[sltb70088-bib-0032] Rasic, D. , J. A. Robinson , J. Bolton , O. J. Bienvenu , and J. Sareen . 2011. “Longitudinal Relationships of Religious Worship Attendance and Spirituality With Major Depression, Anxiety Disorders, and Suicidal Ideation and Attempts: Findings From the Baltimore Epidemiologic Catchment Area Study.” Journal of Psychiatric Research 45, no. 6: 848–854. 10.1016/j.jpsychires.2010.11.014. 21215973

[sltb70088-bib-0033] Salway, T. , L. E. Ross , C. P. Fehr , et al. 2019. “A Systematic Review and Meta‐Analysis of Disparities in the Prevalence of Suicide Ideation and Attempt Among Bisexual Populations.” Archives of Sexual Behavior 48, no. 1: 89–111. 10.1007/s10508-018-1150-6. 29492768

[sltb70088-bib-0034] Satorra, A. , and P. M. Bentler . 2001. “A Scaled Difference Chi‐Square Test Statistic for Moment Structure Analysis.” Psychometrika 66, no. 4: 507–514. 10.1007/BF02296192. PMC2905175 20640194

[sltb70088-bib-0035] Slupphaug, K. S. , M. Mehmetoglu , and M. Mittner . 2024. “Modsem: An R Package for Estimating Latent Interactions and Quadratic Effects.” Structural Equation Modeling: A Multidisciplinary Journal 32: 717–729. 10.1080/10705511.2024.2417409.

[sltb70088-bib-0056] Smith, T. B. , M. E. McCullough , and J. Poll . 2003. “Religiousness and Depression: Evidence for a Main Effect and the Moderating Influence of Stressful Life Events.” Psychological Bulletin (US) 129, no. 4: 614–636. 10.1037/0033-2909.129.4.614. 12848223

[sltb70088-bib-0036] Sowe, B. J. , J. Brown , and A. J. Taylor . 2014. “Sex and the Sinner: Comparing Religious and Nonreligious Same‐Sex Attracted Adults on Internalized Homonegativity and Distress.” American Journal of Orthopsychiatry 84, no. 5: 530–544. 10.1037/ort0000021. 25265218

[sltb70088-bib-0037] Thomas, J. , and M. Barbato . 2020. “Positive Religious Coping and Mental Health Among Christians and Muslims in Response to the COVID‐19 Pandemic.” Religion 11, no. 10: 498. 10.3390/rel11100498.

[sltb70088-bib-0038] Tierney, N. , D. Cook , M. McBain , and C. Fay . 2017. “naniar: Data Structures, Summaries, and Visualisations for Missing Data (p. 1.1.0) [Dataset].” 10.32614/CRAN.package.naniar.

[sltb70088-bib-0039] Todd, N. R. , D. M. Nguyễn , A. M. Blackburn , R. La , and S. Kim . 2025. “Linking the Religious and Social Environment to Sexual Minority Mental Health.” American Journal of Community Psychology 76: 408–422. 10.1002/ajcp.70002. 40685835 PMC12747596

[sltb70088-bib-0040] Todd, N. R. , S. Watanabe , and A. M. Blackburn . 2025. “Examining the Religious Residue Among Racial‐Ethnically Diverse Sexual Minorities.” International Journal for the Psychology of Religion 36: 1–18. 10.1080/10508619.2025.2521571.

[sltb70088-bib-0041] Valen, B. M. , D. Orantes , S. E. Burke , and K. M. Antshel . 2025. “Health Disparities Among Transgender, Nonbinary, and Cisgender Undergraduate Students.” Psychology of Sexual Orientation and Gender Diversity. 10.1037/sgd0000815.

[sltb70088-bib-0042] Van Orden, K. A. , T. K. Witte , K. C. Cukrowicz , S. R. Braithwaite , E. A. Selby , and J. Joiner Jr. 2010. “The Interpersonal Theory of Suicide.” Psychological Review 117, no. 2: 575–600. 10.1037/a0018697. 20438238 PMC3130348

[sltb70088-bib-0043] VanderWeele, T. J. , S. Li , A. C. Tsai , and I. Kawachi . 2016. “Association Between Religious Service Attendance and Lower Suicide Rates Among US Women.” JAMA Psychiatry 73, no. 8: 845–851. 10.1001/jamapsychiatry.2016.1243. 27367927 PMC7228478

[sltb70088-bib-0044] Whitley, B. E., Jr. 2009. “Religiosity and Attitudes Toward Lesbians and Gay Men: A Meta‐Analysis.” International Journal for the Psychology of Religion 19, no. 1: 21–38. 10.1080/10508610802471104.

[sltb70088-bib-0045] Wittgens, C. , M. M. Fischer , P. Buspavanich , S. Theobald , K. Schweizer , and S. Trautmann . 2022. “Mental Health in People With Minority Sexual Orientations: A Meta‐Analysis of Population‐Based Studies.” Acta Psychiatrica Scandinavica 145, no. 4: 357–372. 10.1111/acps.13405. 35090051

[sltb70088-bib-0046] Woodell, B. , and P. Schwadel . 2020. “Changes in Religiosity Among Lesbian, Gay, and Bisexual Emerging Adults.” Journal for the Scientific Study of Religion 59, no. 2: 379–396. 10.1111/jssr.12653.

[sltb70088-bib-0047] Wu, A. , J.‐Y. Wang , and C.‐X. Jia . 2015. “Religion and Completed Suicide: A Meta‐Analysis.” PLoS One 10, no. 6: e0131715. 10.1371/journal.pone.0131715. 26110867 PMC4482518

