# Supplementary material for: Is Religious Coping a Protective Factor, and for Whom? A Moderation and Subgroup Analysis on Loneliness and Suicide Attempts Among LGBTQ+ Adults Raised as Latter‐Day Saints
Source: Suicide Life Threat Behav. 2026 Mar 31;56(2):e70088. doi: 10.1111/sltb.70088 (PMC13036475; doi:10.1111/sltb.70088)
Supplement: Supplementary file 1 — Appendix S1: sltb70088‐sup‐0001‐AppendixS1.docx. [file SLTB-56-0-s001.docx]

**Is religious coping a protective factor, and for whom? A moderation and subgroup analysis on loneliness and suicide attempts among LGBTQ+ adults raised as Latter-Day Saints**

Supplemental Materials

**Table S1.** *Religious Coping by Religious Affiliation*

| Contrast | *b* | *SE* | *df* | *t* ratio | *p* value |
| --- | --- | --- | --- | --- | --- |
| Christian/Catholic - LDS | 0.16 | 0.14 | 365 | 1.10 | 0.688 |
| Christian/Catholic - None | 2.03 | 0.15 | 365 | 13.15 | < 0.001 |
| Christian/Catholic - Other | 1.26 | 0.30 | 365 | 4.11 | < 0.001 |
| LDS - None | 1.87 | 0.10 | 365 | 17.16 | < 0.001 |
| LDS - Other | 1.10 | 0.28 | 365 | 3.84 | < 0.001 |
| None - Other | -0.76 | 0.29 | 365 | -2.62 | 0.045 |

*Note.* Tukey’s post-hoc adjustment was applied. LDS = Latter-day Saints.

**Figure S1.** *Religious Coping by Religious Affiliation*

**
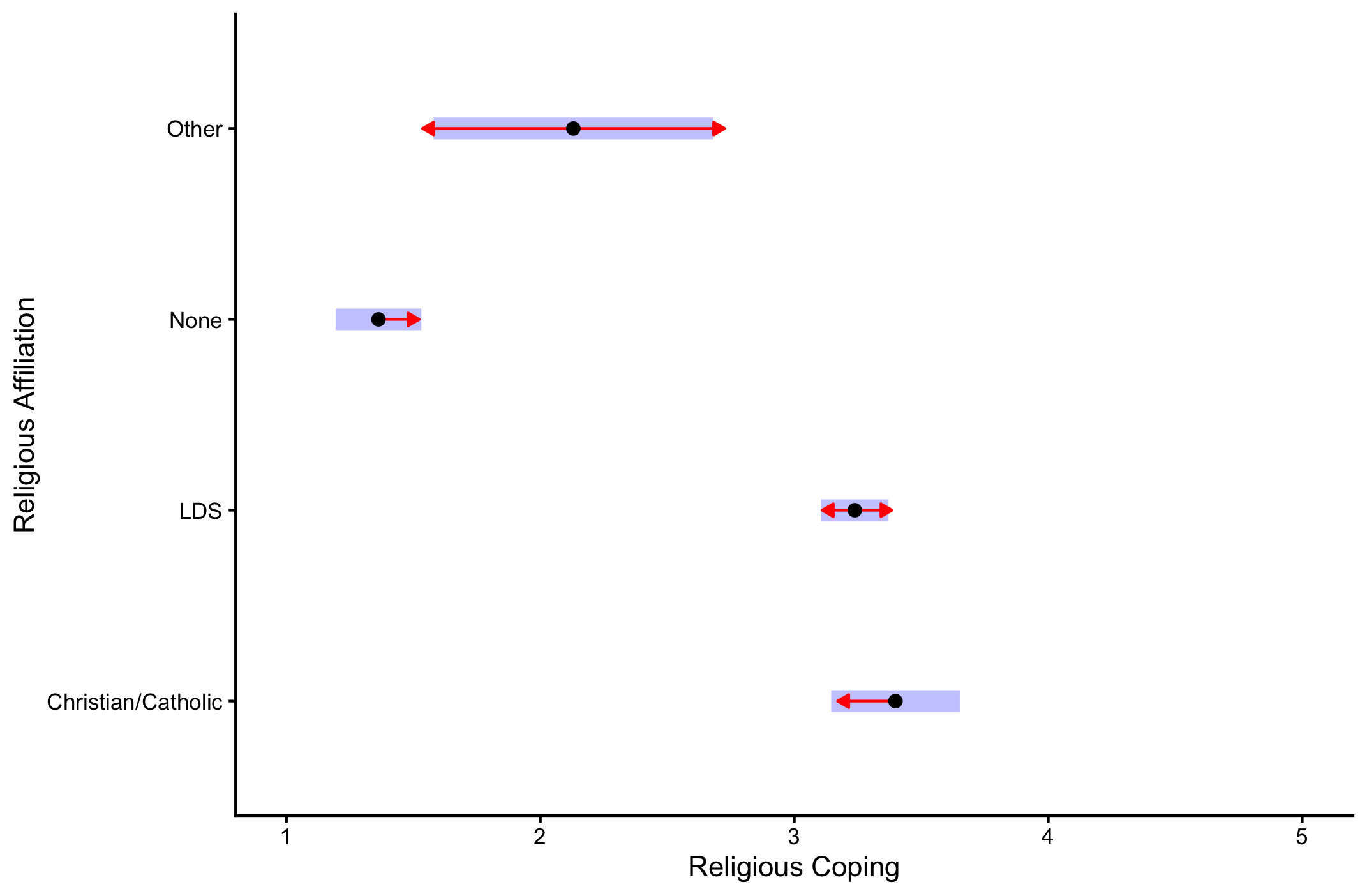
**

*Note.* Non-overlapping arrows suggest significant pairwise differences using Tukey post-hoc corrections. LDS = Latter-day Saints.
